# Comparison between pressure support ventilation and T-piece in spontaneous breathing trials

**DOI:** 10.1186/s12931-022-01942-w

**Published:** 2022-02-07

**Authors:** Soo Jin Na, Ryoung-Eun Ko, Jimyoung Nam, Myeong Gyun Ko, Kyeongman Jeon

**Affiliations:** 1 Department of Critical Care Medicine, Samsung Medical Center, Sungkyunkwan University School of Medicine, Seoul, Republic of Korea; 2 Intensive Care Unit Nursing Department, Samsung Medical Center, Sungkyunkwan University School of Medicine, Seoul, Republic of Korea; 3 Division of Pulmonary and Critical Care Medicine, Department of Medicine, Samsung Medical Center, Sungkyunkwan University School of Medicine, 81 Irwon-ro, Gangnam-gu, Seoul, 06351 Republic of Korea

**Keywords:** Spontaneous breathing trial, T-piece, Pressure support ventilation, Ventilator weaning

## Abstract

**Background:**

Recent guidelines recommended conducting spontaneous breathing trial (SBT) with modest inspiratory pressure augmentation rather than T-piece or continuous positive airway pressure. However, it was based on few studies focused on the outcomes of extubation rather than the weaning process, despite the existence of various weaning situations in clinical practice. This study was designed to investigate the effects of SBT with pressure support ventilation (PSV) or T-piece on weaning outcomes.

**Methods:**

All consecutive patients admitted to two medical intensive care units (ICUs) and those requiring mechanical ventilation (MV) for more than 24 h from November 1, 2017 to September 30, 2020 were prospectively registered. T-piece trial was used until March 2019, and then, pressure support of 8 cmH_2_O and 0 positive end-expiratory pressure were used for SBT since July 2019, after a 3-month transition period for the revised SBT protocol. The primary outcome of this study was successful weaning defined according to the WIND (Weaning according to a New Definition) definition and were compared between the T-piece group and PSV group. The association between the SBT method and weaning outcome was evaluated with logistic regression analysis.

**Results:**

In this study, 787 eligible patients were divided into the T-piece (n = 473) and PSV (n = 314) groups after excluding patients for a 3-month transition period. Successful weaning was not different between the two groups (85.0% vs. 86.3%; *p* = 0.607). However, the PSV group had a higher proportion of short weaning (70.1% vs. 59.0%; *p* = 0.002) and lower proportion of difficult weaning (13.1% vs. 24.1%; *p* < 0.001) than the T-piece group. The proportion of prolonged weaning was similar between the two groups (16.9% vs. 16.9%; *p* = 0.990). After excluding patients who underwent tracheostomy before the SBTs, similar results were found. Reintubation rates at 48 h, 72 h, and 7 days following the planned extubation were not different between the PSV and T-piece groups. Moreover, no significant differences in intensive care unit and hospital mortality and length of stay were observed.

**Conclusions:**

In critically ill medical patients, SBT using PSV was not associated with a higher rate of successful weaning compared with SBT using T-piece. However, PSV could shorten the weaning process without increasing the risk of reintubation.

**Supplementary Information:**

The online version contains supplementary material available at 10.1186/s12931-022-01942-w.

## Background

Patients experiencing delayed extubation and prolonged mechanical ventilation (MV) are associated with an increased risk of ventilator-associated pneumonia and increased length of stay and mortality [1]. Alternatively, extubation in patients not yet ready to be liberated from MV requires reintubation, which is also associated with increased morbidity and mortality [2–4]. Therefore, to objectively assess the readiness of a patient to maintain spontaneous breathing without MV support, a spontaneous breathing trial (SBT), which is a method for evaluating a patient’s respiratory function for a certain period under a similar work of breathing after extubation, is commonly recommended [5].

Recent guidelines recommended conducting SBT with modest inspiratory pressure augmentation rather than without inspiratory pressure support, such as T-piece [6]. However, it was based on few studies focused on the outcomes of extubation rather than the weaning process, despite the existence of various weaning situations in clinical practice. Moreover, it conforms to the limitations of an International Consensus Conference (ICC) classification, a method for evaluating weaning outcomes that can only be used in patients with endotracheal tube [7]. Researchers in the Weaning according to a New Definition (WIND) study have suggested a new classification that overcomes the limitations of the ICC classification [8]. Recently, we reported that the WIND classification applies to all mechanically ventilated patients, regardless of the type of artificial airway, and has a higher discriminatory power for weaning outcomes [9].

Therefore, in this study, we investigated the effects of inspiratory pressure augmentation during SBT on weaning outcomes based on the WIND classification in medical patients receiving MV and compared them with those of T-piece.

## Methods

### Study population

**Fig. 1:**
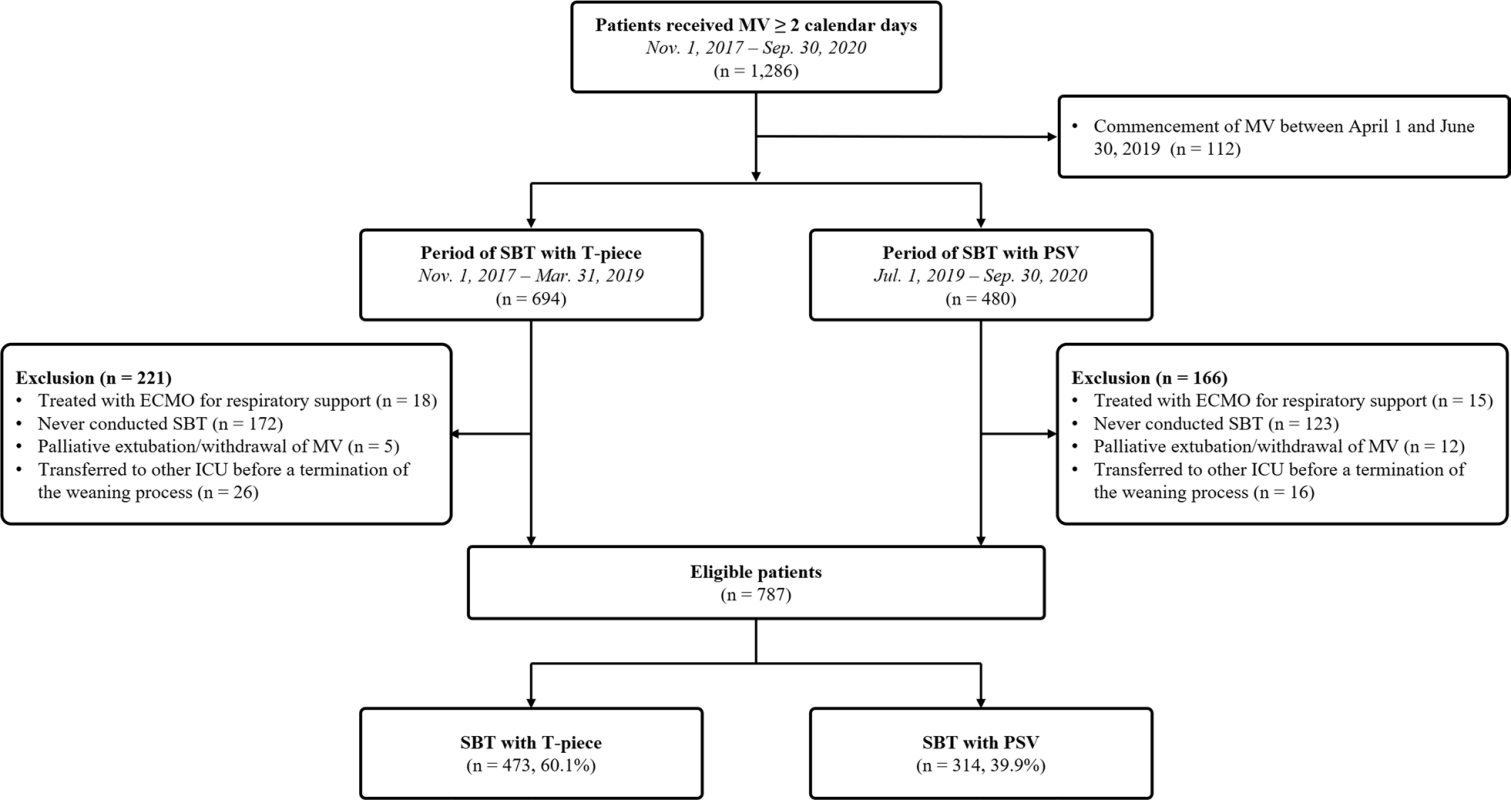
Scheme of group distribution. *MV* mechanical ventilation, *ECMO* extracorporeal membrane oxygenation, *SBT* spontaneous breathing trial, *ICU* intensive care unit, *PSV* pressure support ventilation

Data were obtained from the ongoing prospective observational study on the assessment of process and outcome of protocol-based weaning from MV in the medical patients (ClinicalTrials.gov identifier: NCT05134467), which began in November 2017. All consecutive patients admitted to two medical intensive care units (ICUs) and those requiring MV for more than 24 h from November 2017 were prospectively registered at Samsung Medical Center (a 1989-bed tertiary referral hospital with tertiary-level ICUs) in Seoul, South Korea. In the two medical ICUs, general critical care was provided based on the same principle and protocols by multidisciplinary teams. Patients aged 19 years and older who received MV for at least 2 calendar days between November 1, 2017 and September 30, 2020 were considered eligible, and 1286 patients were identified. Among them, we excluded 112 patients who received MV support between April 1, 2019 and June 30, 2019, which is a 3-month transition period to SBT with pressure support ventilation (PSV) from T-piece, to avoid the inclusion of mixed patients who underwent SBT with T-piece and/or PSV during their weaning process. Eligible patients were divided into the T-piece (before April 2019) and PSV (after July 2019) groups according to the date of initiation of MV (Fig. 1).

The Institutional Review Board of Samsung Medical Center approved this study (SMC 2017-08-141-009) and waived the requirement for informed consent because of the observational nature of the study. Additionally, the patients’ data were anonymized and de-identified before the analysis.

### Process of weaning from MV

Our hospital implemented standardized weaning programs using a respiratory care practitioner-driven, protocol-directed approach since 2010. The details of the weaning process have been described in previous publications [9–11]. An additional file shows this in more detail (see Additional file 1). The criteria for weaning readiness included the resolution of the acute phase of the disease for which the patient was intubated, adequate coughing, the absence of excessive tracheobronchial secretion, stable cardiovascular status, adequate oxygenation, adequate pulmonary function, and adequate mentation. If the patient fulfilled the criteria of readiness for weaning trial, they underwent the SBT according to the protocol.

Before April 2019, SBT was performed using T-piece for all patients who met the criteria for readiness to wean. MV was disconnected from the patient, and supplemental oxygen was provided as a blended gas at a flow of 9 L/min with less than 40% of inspired oxygen fraction through the T-piece system connected to the endotracheal or tracheostomy tube. In April 2019, our hospital revised the weaning protocol for conducting SBT using inspiratory pressure augmentation rather than T-piece in patients with an endotracheal tube based on recent guidelines [6], and this novel protocol was implemented in selected patients for a 3-month transition period for feasibility. Then, from July 1, 2019, the revised protocol using inspiratory pressure augmentation for SBT was implemented to all patients who met the criteria for readiness to wean. The patients underwent SBT while attached to the ventilator settings with pressure support of 8 cmH_2_O and PEEP of 0. FiO_2_ maintained the same as before the SBT. The initial attempt was targeted at 30 min for both the T-piece trial and inspiratory pressure augmentation, but the trial was immediately terminated when a sign of SBT failure occurred. The patients’ blood pressure, heart rates, respiratory rates, and transcutaneous oxygen saturation were continuously monitored during the trial. When the trial was terminated, arterial blood was obtained for blood gas analysis, and the patient returned to MV with the same ventilator settings as those before the SBT.

### Data collection and clinical outcomes

Clinical, laboratory, and outcome data were prospectively collected by a trained study coordinator. The demographics of the patients and major cause of intubation were evaluated and recorded by the physicians on the day of MV support initiation. Details of the patients’ weaning readiness and SBT were recorded in a specified format on the day of the assessment by respiratory care practitioners. The values of the MV setting and respiratory parameters were synchronized to the hospital electronic medical chart and recorded every hour, and we collected the values at 8 am on the day of the first SBT.

The primary outcome of this study was successful weaning defined according to the WIND definition as extubation without death or reintubation within the next 7 days of SBT or ICU discharge without invasive MV within 7 days, whichever comes first for intubated patients and as spontaneous ventilation through tracheostomy without any MV during 7 consecutive days or discharged with spontaneous breathing, whichever comes first for patients with tracheostomy after the first separation attempt [8]. The date of successful weaning was recorded to the actual day of extubation or spontaneous ventilation through tracheostomy after the patient had completed 7 days of SBT without reintubation or any MV through tracheostomy (or was alive and discharged earlier). The secondary outcomes included the WIND classification of weaning, reintubation among patients who were extubated, the incidence of tracheostomy after the first separation attempt, length of stay, and mortality. We classified weaning using the WIND classification, which grouped the patients according to the duration between the first SBT and weaning termination [8]: in group 1 (short weaning), the first attempt resulted in the termination of the weaning process within 1 day; in group 2 (difficult weaning), the weaning process was completed after more than 1 day but in less than 1 week after the first separation attempt; in group 3 (prolonged weaning), the weaning process was still not terminated 7 days after the first separation attempt.

Sensitivity analysis was performed only with patients who underwent the first SBT using endotracheal tube to exclude possible bias of tracheostomy [12].

### Statistical analysis

Descriptive statistics were performed to compare the clinical characteristics and weaning outcomes between the T-piece and PSV groups. Continuous variables were expressed as medians and interquartile ranges (IQRs) and examined using the Mann–Whitney U-test. Categorical variables were presented as numbers and percentages and were analyzed using the Chi-square test or Fisher’s exact test, where applicable. To adjust for potential confounding factors in the association between SBT using PSV and weaning outcomes, logistic regression analysis was used. Variables with a p-value < 0.1 on univariate analyses, as well as a priori variables that were clinically relevant, were entered into the forward stepwise multiple logistic regression model. Data are presented as odds ratios (ORs) with 95% confidence interval (CI). And then, we conducted further analyses to assess the effectiveness of SBT using PSV across subgroups, which were specified according to the patient's underlying disease and major reason for MV. A two-tailed *p*-value of less than 0.05 was considered statistically significant for all analyses. Data were analyzed using STATA version 16.0 (Stata Corp., College Station, TX, USA).

## Results

### Baseline demographic and clinical characteristics

Among the 787 eligible patients, 473 used T-piece and 314 patients used PSV as the initial SBT. The characteristics of the patients in the T-piece and PSV groups are shown in Table 1. No significant differences in age, sex, and the major reason for MV were observed between the two groups. However, the rates of heart failure and myopathies/neuropathies were higher in the PSV group than those in the T-piece group. The most common cause of intubation was hypoxemic respiratory failure (33.0%), followed by hypercapnic respiratory failure (26.6%) and shock (25.4%). Hypoxemic respiratory failure was the most common cause in the two groups.

**Table 1:** Baseline demographic and clinical characteristics (N = 787)

	T-piece group (n = 473)	PSV group (n = 314)	*p*-value
Age, years	67 (57–75)	67 (57–76)	0.726
Male	307 (64.9)	209 (66.56)	0.632
Body mass index, kg/m^2^	22.2 (19.7–25.3)	22.7 (19.7–25.8)	0.523
Comorbidities			
Chronic obstructive pulmonary disease	33 (7.9)	26 (9.5)	0.448
Asthma	8 (1.9)	5 (1.3)	0.941
Interstitial lung disease	9 (2.2)	10 (3.7)	0.233
Heart failure: NYHA classes III–IV	31 (7.4)	37 (13.6)	0.008
Chronic renal failure	61 (14.6)	45 (16.5)	0.492
Liver cirrhosis: Child–Pugh Class C	12 (2.9)	9 (3.3)	0.746
Solid/hematologic malignancy	263 (62.8)	169 (61.9)	0.819
Myopathies/Neuropathies	30 (7.2)	36 (13.2)	0.008
Dementia	142 (30.0)	90 (28.7)	0.682
Major reason for MV			0.069
Hypoxemic respiratory failure	159 (33.6)	101 (32.2)	
Hypercapnic respiratory failure	140 (29.6)	69 (22.0)	
Shock	111 (23.5)	89 (28.3)	
Surgery	5 (1.1)	6 (1.9)	
Others^a^	58 (12.3)	49 (15.6)	

Values are median with interquartile range or number (%)

*MV* mechanical ventilation, *NYHA* New York Heart Association, *PSV* pressure support ventilation

^a^Others include airway protection, neurological impairment, and metabolic causes

### Patient characteristics on the day of the first SBT

The first SBT was performed on the median of 5 days after the commencement of MV in both groups (*p* = 0.225) (Table 2). No significant differences in the use of analgesics and sedatives and the RASS score were observed between the two groups. The use of vasoactive drugs (19.2% vs. 8.5%; *p* < 0.001) and opioids (78.6% vs. 69.4%; *p* = 0.004) was higher in the T-piece group than that in the PSV group, but the use of steroids, diuretics, and renal replacement therapy was not different. On the day of the first SBT, the median sequential organ failure assessment (SOFA) score was 7 (IQR, 4–9) and 7 (IQR, 5–10), respectively (*p* = 0.197). Most patients had pressure support ventilation, and the MV settings and results of arterial blood gas analysis on the day of the first SBT are presented in Table 2.

**Table 2:** Patient characteristics on the day of the first spontaneous breathing trial (N = 787)

	T-piece group (n = 473)	PSV group (n = 314)	*p*-value
Duration of MV before first spontaneous breathing trial, days	5 (3–7)	5 (4–7)	0.225
Weak cough capacity	67 (18.9)	49 (19.8)	0.799
Abundant tracheal secretions	25 (7.1)	17 (6.9)	0.922
Artificial airway			0.220
Endotracheal tube	375 (79.3)	260 (82.8)	
Tracheostomy	98 (20.7)	54 (17.2)	
Medical management			
Vasoactive drug	87 (19.2)	26 (8.5)	< 0.001
Sedatives	190 (41.9)	138 (45.0)	0.397
Opioid	357 (78.6)	213 (69.4)	0.004
Steroid	212 (46.7)	137 (44.6)	0.574
Diuretics	178 (39.2)	113 (36.8)	0.504
Renal replacement therapy	57 (12.6)	55 (17.9)	0.041
RASS score			0.659
RASS − 1–+ 1	332 (73.3)	232 (75.6)	
RASS < − 1	106 (23.4)	68 (22.2)	
RASS > + 1	15 (3.3)	7 (2.3)	
SOFA scores	7 (4–9)	7 (5–10)	0.197
Setting of MV			
Mode			0.251
Volume controlled ventilation	0 (0.0)	1 (0.3)	
Pressure controlled ventilation	48 (10.6)	27 (8.8)	
Synchronized intermittent mandatory ventilation	0 (0.0)	1 (0.3)	
Pressure support ventilation	406 (89.4)	278 (90.6)	
Peak inspiratory pressure, cmH_2_O	16 (14–22)	16 (14–18)	0.142
Respiratory rate, breath/min	18 (14–19)	18 (14–23)	0.258
PEEP, cmH_2_O	5 (5–5)	5 (5–5)	0.266
Monitored Vt/PBW, mL/kg	8.1 (6.3–10.1)	7.1 (5.8–9.2)	0.001
FiO_2_, %	30 (30–40)	30 (25–40)	0.003
PaO_2_/FiO_2_ ratio	288 (223–375)	300.5 (230–391.7)	0.135
Arterial blood gas			
pH	7.457 (7.420–7.494)	7.471 (7.436–7.500)	0.008
PaCO_2_, mmHg	35.5 (31.0–41.9)	33.9 (29.2–40.2)	0.063
PaO_2_, mmHg	91.8 (78.9–106.1)	90.0 (79.9–103.2)	0.790
SaO_2_, %	97.0 (95.6–98.4)	97.0 (95.7–98.0)	0.149
Lactate, mmol/L	1.69 (1.14–2.32)	2.01 (1.26–2.46)	0.263

Values are median with interquartile range or number (%)

*FiO*_*2*_ fraction of inspired oxygen, *MV* mechanical ventilation, *PaCO*_*2*_ partial pressure of carbon dioxide in arterial blood, *PaO*_*2*_ partial pressure of oxygen in arterial blood, *PBW* predicted body weight, *PEEP* positive end-expiratory pressure, *PSV* pressure support ventilation, *SaO*_*2*_ arterial oxygen saturations, *SOFA* Sequential Organ Failure Assessment, *RASS* Richmond Agitation–Sedation Scale, *VT* tidal volume

### Clinical outcomes

**Fig. 2:**
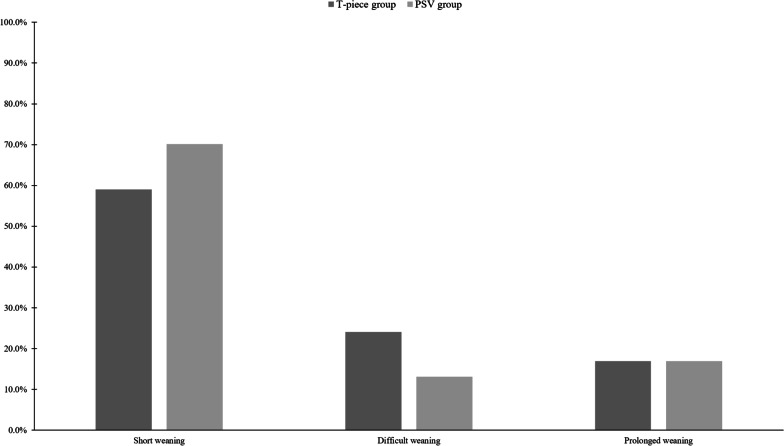
Comparisons of weaning outcomes by WIND classification. Bars show the proportion of patients with short, difficult, or prolonged weaning based on the duration between the first SBT and weaning termination according to the initial SBT performed using T-piece (black bars) or PSV (gray bars)

Of the 787 patients, 673 (85.5%) successfully weaned from MV (Table 3). No significant difference in the successful weaning (85.0% vs. 86.3%; *p* = 0.607) was observed; however, the duration from the first SBT to the final liberation from MV in patients with successful weaning was statistically shorter in the PSV group (median, 0 days; IQR, 0–0 days) than in the T-piece group (median, 0 days; IQR, 0–2 days) (*p* = 0.002). The PSV group had a higher proportion of patients with short weaning (70.1% vs. 59.0%; *p* = 0.002) and lower proportion of patients with difficult weaning (13.1% vs. 24.1%; *p* < 0.001) than the T-piece group (Fig. 2). The proportion of patients with prolonged weaning was similar between the two groups (16.9% vs. 16.9%; *p* = 0.990). In addition, proportion of prolonged weaning according to the comorbidities was not different between the two groups, but higher rate of prolonged weaning in PSV group than T-piece group (29.7% vs. 9.7%; *p* = 0.042) in patients with heart failure (Additional file 2: Fig. S1). Finally, the duration of the weaning process was shorter in the PSV group (median, 0 days; IQR, 0–3 days) than in the T-piece group (median, 0 days; IQR, 0–4 days) (*p* = 0.022). However, ICU and hospital mortality and length of stay were not different between the two groups (Table 3).

**Table 3:** Clinical outcomes (N = 787)

	T-piece group (n = 473)	PSV group (n = 314)	*p*-value
Successful weaning	402 (85.0)	271 (86.3)	0.607
Duration from the 1st SBT to the final liberation from MV	0 (0–2)	0 (0–0)	0.002
WIND classification			
Short weaning	279 (59.0)	220 (70.1)	0.002
Difficult weaning	114 (24.1)	41 (13.1)	< 0.001
Prolonged weaning	80 (16.9)	53 (16.9)	0.990
Duration of weaning process, days	0 (0–4)	0 (0–3)	0.022
Tracheostomy	151 (31.9)	96 (30.6)	0.689
Before the 1st SBT	98 (64.9)	54 (56.3)	
After the 1st SBT	53 (35.1)	42 (43.8)	
Mortality			
Intensive care unit	56 (11.8)	34 (10.8)	0.662
Hospital	142 (30.0)	63 (30.7)	0.853
Length of stay			
Intensive care unit, days	8 (6–15)	9 (5–14)	0.328
Hospital, days	31 (17–55)	26 (15–45)	0.118

Values are median with interquartile range or number (%)

*PSV* pressure support ventilation, *WIND* Weaning according to a New Definition

The results of univariable and multivariable analyses with the logistic regression model for probability of successful weaning are presented in Table 4. After adjusting for potential confounding factors, steroid use (adjusted OR 0.47, 95% CI 0.24–0.92, *p* = 0.027), tracheostomy (adjusted OR 0.38, 95% CI 0.16–0.90, *p* = 0.027), and SOFA score except respiratory system on the day of the first SBT (adjusted OR 0.87, 95% CI 0.78–0.96, *p* = 0.007) were independently associated with decreased rate of successful weaning (Table 4). However, SBT using PSV was not associated with successful weaning. No significant interaction was noted between comorbidities or reason for MV and SBT group with respect to the primary outcome (Additional file 3: Fig. S2).

**Table 4:** Univariable and multivariable analyses with logistic regression model for probability of successful weaning (N = 787)

	Univariable			Multivariable		
	OR	95% CI	*p*-value	Adjusted OR	95% CI	p-value
SBT with PSV	1.11	0.74–1.68	0.608	0.64	0.32–1.27	0.199
Chronic renal failure	1.78	0.92–3.44	0.088	2.42	0.75–7.81	0.138
Solid/hematologic malignancy	0.63	0.40–0.98	0.039	0.75	0.35–1.59	0.451
Steroid use	0.60	0.40–0.90	0.013	0.47	0.24–0.92	0.027
RASS score^a^						
RASS < − 1	0.38	0.25–0.58	< 0.001	0.77	0.35–1.67	0.509
RASS > + 1	0.81	0.23–2.82	0.741	0.35	0.06–2.09	0.250
Duration of MV before first SBT, days	0.92	0.88–0.96	< 0.001	0.96	0.89–1.04	0.357
Weak cough capacity	0.34	0.21–0.57	< 0.001	0.51	0.25–1.07	0.074
Tracheostomy	0.33	0.22–0.52	< 0.001	0.38	0.16–0.90	0.027
Peak inspiratory pressure, cmH_2_O	0.91	0.86–0.95	< 0.001	0.90	0.81–1.01	0.069
Respiratory rate, breath/min	0.96	0.94–0.99	0.011	1.00	0.95–1.05	0.881
PaCO_2_, mmHg	0.98	0.96–1.00	0.077	1.00	0.96–1.04	0.912
PaO_2_/FiO_2_ ratio	1.00	1.00–1.00	0.033	1.00	1.00–1.00	0.501
SOFA score except respiratory system	0.88	0.83–0.93	< 0.001	0.87	0.78–0.96	0.007

*CI* confidence interval, *FiO*_*2*_ fraction of inspired oxygen, *MV* mechanical ventilation, *OR* odds ratio, *PaCO*_*2*_ partial pressure of carbon dioxide in arterial blood, *PaO*_*2*_ partial pressure of oxygen in arterial blood, *PSV* pressure support ventilation, *RASS* Richmond Agitation-Sedation Scale, *SBT* spontaneous breathing trial, *SOFA* sequential organ failure assessment

^a^The reference group is a RASS − 1–+ 1

### Sensitivity analysis

The baseline demographic and clinical characteristics of the two groups with only patients with endotracheal tube who underwent the first SBT are shown in Additional file 4: Table S1. No significant differences were observed between the two groups; however, the PSV group had a higher rate of heart failure than the T-piece group (13.4% vs. 7.9%; *p* = 0.036). The most common cause of intubation was hypoxemic respiratory failure in the T-piece group (32.3%); however, shock was the most common cause of intubation in the PSV group (31.5%). The characteristics of the patients on the day of the first SBT are presented in Additional file 4: Table S2. The duration of MV before the first SBT was 4 days (IQR, 3–6 days) in the T-piece group and 5 days (IQR, 3–7 days) in the PSV group (*p* = 0.026). However, the other characteristics were similar to the results of our main analysis.

Among them, 500 (78.7%) patients were successfully extubated and never underwent tracheostomy, 22 (3.5%) patients were not extubated and underwent tracheostomy after SBT failure using an endotracheal tube. The remaining 107 (16.9%) patients were extubated but experienced reintubation and then tracheostomy due to extubation failure before the final liberation from MV. In addition, 6 (0.9%) patients maintained the endotracheal tube until death. The PSV group had a higher proportion of patients with short weaning (76.5% vs. 66.7%; *p* = 0.007) and lower proportion of patients with difficult weaning (9.6% vs. 20.5%; *p* < 0.001) than the T-piece group (Table 5). The proportion of patients with prolonged weaning was similar between the two groups (13.9% vs. 12.8%; *p* = 0.702). Finally, the duration of the weaning process was shorter in the PSV group (median, 0; IQR, 0–3 days) than that in the T-piece group (median, 0 days; IQR, 0–4 days) (*p* = 0.022). However, the reintubation rates at 48 h (16.5% vs. 16.5%), 72 h (17.7% vs. 16.8%), and 7 days (18.1% vs. 17.0%) following the planned extubation were not different between the two groups (Table 5).

**Table 5:** Clinical outcomes of patient who underwent the first SBT using endotracheal tube (n = 635)

	T-piece group (n = 375)	PSV group (n = 260)	*p*-value
Successful weaning	332 (88.5)	231 (88.9)	0.903
Duration from 1st SBT to final liberation from MV, days	0 (0–1)	0 (0–0)	0.005
WIND classification			
Short weaning	250 (66.7)	199 (76.5)	0.007
Difficult weaning	77 (20.5)	25 (9.6)	< 0.001
Prolonged weaning	48 (12.8)	36 (13.9)	0.702
Duration of weaning process, days	0 (0–4)	0 (0–3)	0.022
Reintubation			
Within 2 days	59 (16.5)	41 (16.5)	0.996
Within 3 days	60 (16.8)	44 (17.7)	0.770
Within 7 days	61 (17.0)	45 (18.1)	0.742
Overall	62 (17.3)	45 (18.1)	0.811
Tracheostomy	53 (14.1)	42 (16.2)	0.483
Mortality			
Intensive care unit	35 (9.3)	24 (9.2)	0.965
Hospital	104 (27.7)	55 (31.4)	0.373
Length of stay			
Intensive care unit, days	8 (6–11)	8 (5–12)	0.828
Hospital, days	25 (15–45)	25 (14–42)	0.816

Values are median with interquartile range or number (%)

*PSV* pressure support ventilation, *SBT* spontaneous breathing trial, *WIND* Weaning according to a New Definition

## Discussion

This study evaluated the differences in weaning outcomes between SBT using PSV and SBT using T-piece based on the WIND classification in medical patients receiving MV. SBT using PSV was not associated with a higher rate of successful weaning compared with SBT using T-piece. Additionally, no difference in the length of stay and mortality between the two groups. However, the PSV group had a significantly higher rate of short weaning than the T-piece group, and this result was maintained only in patients who underwent the first SBT using endotracheal tube without increasing the risk of reintubation.

Several studies have attempted to develop a weaning strategy that can reliably identify a patient’s readiness to be weaned from ventilator support. Our standardized weaning protocol, led by respiratory care practitioners and consisting of a readiness test and SBT using a set of objective parameters, is in this context [13]. Several methods are currently being used for SBT, although considerable debate exists regarding the optimal SBT method that simulates a patient’s work of breathing after extubation. The T-piece trial, one of the methods we used, is the simplest form of SBT and more accurately reflects the physiological conditions after extubation [14, 15]. However, the airway resistance inherent in the endotracheal tube raises concerns about the work of breathing during the SBT [16]. Therefore, minimal inspiratory pressure augmentation is often chosen during SBT to compensate for the work imposed by the endotracheal tube. Sklar et al. have compared respiratory effort among the SBT methods through physiological meta-analysis and demonstrated that PSV reduces work of breathing and pressure–time product compared with other SBT methods [14]. The advantage of PSV is that it can increase the probability of liberation from MV in certain patients. Ezingeard et al. have shown that some patients who failed the T-piece trial could be successfully extubated after a trial using PSV [17]. Additionally, the PSV was associated with increased successful extubation and decreased duration of MV [18, 19]. Although it is possible that inspiratory pressure support during SBT overestimates the patient’s breathing ability, however, PSV did not increase the rate of extubation failure compared with the T-piece method in practice [19–21]. Based on the results of these studies, the American College of Chest Physicians/American Thoracic Society Clinical Practice Guidelines suggest inspiratory pressure augmentation as an initial SBT for patients who received MV for more than 24 h [6].

However, SBT does not predict well the consequences of tube removal in terms of upper airway patency, lower airway protection, and removal of secretions for the ability to sustain spontaneous breathing. Nonetheless, most studies on SBT methods have evaluated successful extubation than successful SBT and duration of the weaning process, despite the existence of various weaning situations in clinical practice, such as tracheostomized patients [17, 19, 21]. The increase in the work of breathing during the SBT caused by the presence of an endotracheal tube may be an excessive load for some patients breathing through the tube circuit, and poor tolerance of the trial can result in longer MV duration. Several studies have shown that pressure support compensates for the additional work imposed by the endotracheal tube and then reduces external respiratory work and oxygen consumption by respiratory muscles during SBTs [14, 22, 23]. Therefore, SBT using PSV may shorten the weaning process but increase the risk of reintubation following extubation by underestimating the work of breathing needed to breathe without ventilator assistance [24]. However, in a recent meta-analysis [25], the reintubation rate was not significantly different between SBT with PSV and SBT with T-piece, which is consistent with our findings. In this study, the PSV group had a significantly higher rate of short weaning than the T-piece group, and this result was maintained in only patients who underwent the first SBT using endotracheal tube without increasing the risk of reintubation. This is consistent with the recent post-hoc analysis of the previous trial, showing that the proportion of patients who succeeded in the initial SBT was higher using PSV than that using T-piece [26]. Therefore, SBT using PSV might shorten the weaning process without increasing the risk of reintubation. However, a further large prospective clinical trial is needed to confirm these findings.

Although we provided information on the effects of PSV during SBT on weaning outcomes based on the WIND classification, which can encompass various weaning situations and show better ability to predict weaning outcomes than the ICC classification [8, 9, 27], this study has limitations that should be acknowledged. First, given the observational nature of this study, selection bias may have influenced the significance of its findings. Additionally, this study was conducted in a single tertiary care center, which may limit the external validity and generalizability of the findings to other centers because staffing, general critical care management, and the weaning process are different between centers. Second, the influence of the time difference cannot be excluded because the two groups were managed during two periods. However, weaning was performed based on the protocol of our hospital, and no change in the protocol was implemented, except for the SBT technique during the study period. Third, since the study cohort included medical patients with various etiologies of respiratory failure and underlying disease, caution should be taken in applying our results to patients with increased airway resistance. In addition, patients with heart failure or myopathy/neuropathy were higher in the PSV group, which are associated with prolonged weaning [28], and could have an effect on the duration of weaning process. Finally, 30 min for the initial SBT and 120 min for subsequent SBTs were set as the period for evaluating patients in this study; however, the appropriate duration for evaluation has not been defined yet. However, the 30-min trial showed an ability to predict successful extubation comparable to that of the 120-min trial [29, 30].

## Conclusion

In critically ill medical patients, SBT using PSV was not associated with a higher rate of successful weaning compared with SBT using T-piece. However, PSV could shorten the weaning process without increasing the risk of reintubation. A further large prospective randomized controlled trial is needed to confirm these findings in patients with various respiratory pathophysiology and comorbidities before applying this weaning strategy.

## Supplementary Information

**Additional file 1.** Process of weaning from mechanical ventilation at Samsung Medical Center, Seoul, South Korea.

**Additional file 2: Figure S1.** Proportion of prolonged weaning according to comorbidities.

**Additional file 3: Figure S2.** Interactions and odds ratios for successful weaning by subgroups.

**Additional file 4: Table S1.** Baseline demographic and clinical characteristics of the patient who underwent the first SBT using endotracheal tube (n = 635). **Table S2. **Characteristics at the day of first spontaneous breathing trial of patient who performed first SBT using endotracheal tube (n = 635).

## Data Availability

The data that support the findings of this study are available on request from the corresponding author. The data are not publicly available due to privacy or ethical restrictions.

## Abbreviations

FiO_2_: Fraction of inspired oxygen
ICU: Intensive care unit
ICC: International Consensus Conference
MV: Mechanical ventilation
SBT: Spontaneous breathing trial
PEEP: Positive end-expiratory pressure
PSV: Pressure support ventilation
WIND: Weaning according to a New Definition
