# Supplementary material for: Comparison between pressure support ventilation and T-piece in spontaneous breathing trials
Source: Respir Res. 2022 Feb 7;23:22. doi: 10.1186/s12931-022-01942-w (PMC8822807; doi:10.1186/s12931-022-01942-w)
Supplement: Supplementary file 1 — Additional file 1. Process of weaning from mechanical ventilation at Samsung Medical Center, Seoul, South Korea. [file 12931_2022_1942_MOESM1_ESM.docx]

Additional file 1

**Comparison between pressure support ventilation and T-piece in spontaneous breathing trials**

Soo Jin Na, Ryoung-Eun Ko, Jimyoung Nam, Myeong Gyun Ko, Kyeongman Jeon^,^

**Process of weaning from mechanical ventilation**


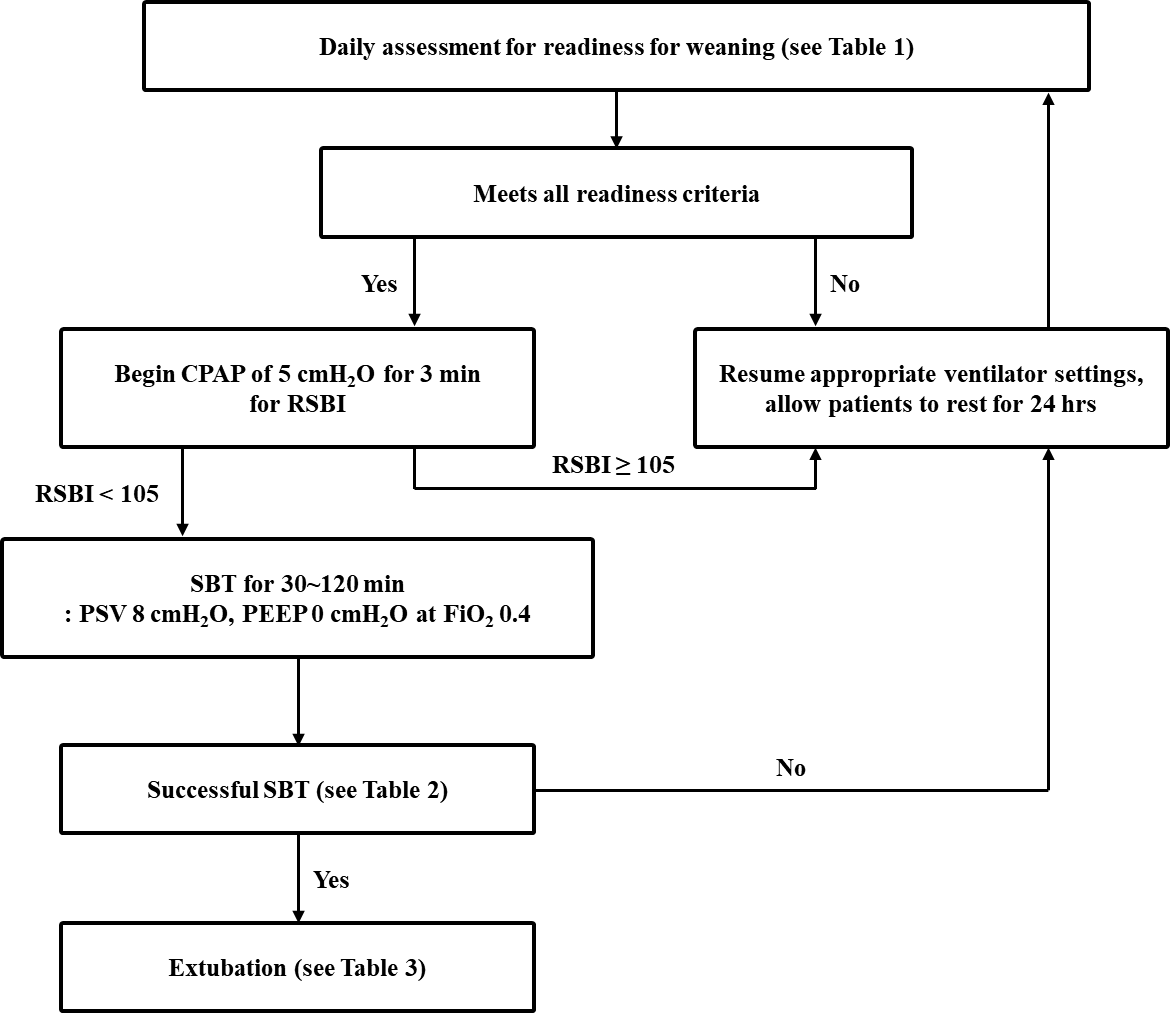


Our hospital implemented standardized weaning programs using a respiratory care practitioner-driven, protocol-directed approach since 2010. Every morning, respiratory care practitioners screened all patients receiving MV for more than 24 h for readiness to wean. The criteria for weaning readiness included the resolution of the acute phase of the disease for which the patient was intubated, adequate coughing, the absence of excessive tracheobronchial secretion, stable cardiovascular status (i.e., heart rate of ≤ 120 beats/min, systolic blood pressure of 90–140 mmHg, and no or minimal vasopressors), adequate oxygenation (i.e., arterial oxygen saturations of more than 90% on fraction of inspired oxygen (FiO_2_) ≤ 0.4 and positive end-expiratory pressure (PEEP) ≤ 5 cmH_2_O), adequate pulmonary function (i.e., respiratory rate of ≤ 35 breathes/min, tidal volume of more than 5 mL/kg, negative inspired pressure ≤ −15 cmH_2_O, rapid shallow breath index of less than 105, and no significant respiratory acidosis), and adequate mentation (no sedation or adequate mentation on sedation). If the patient fulfilled the criteria of readiness for weaning trial (Table 1), they underwent the SBT according to the protocol.

| **Table 1. Considerations for assessing readiness for weaning** |
| --- |
| Clinical assessment   - Adequate cough - Absence of excessive tracheobronchial secretion - Resolution of the underlying cause for respiratory failure |
| Objective measurements   - Adequate mentation   - No sedation or adequate mentation on sedation - Clinical stability   - Stable cardiovascular status: heart rates ≤ 120 beats/min, systolic blood pressure 90-140 mmHg, no or minimal vasopressors use (dopamine or dobutamine 5 mcg/kg/min, norepinephrine 0.05 mcg/kg/min)   - Stable metabolic status - Adequate oxygenation   - SaO_2_ > 90% on FiO_2_ ≤0.4 (or PF ratio > 200)   - PEEP ≤ 5 cmH_2_O - Adequate pulmonary function   - Respiratory rates < 35 breaths/min   - Negative inspired pressure (NIP)^a^ ≤ -15 cmH_2_O   - VT > 5 mL/kg   - VE < 10-15 L/min   - Rapid shallow breath index (RSBI)^b^ < 105   - No significant respiratory acidosis |
| a. NIP: the lowest airway pressure measured during voluntary inspiration with an end-expiratory pause of 20 seconds  b. RSBI: Respiratory rates (in breaths/min) / Tidal volume (in liters) |

SBT was performed using T-piece for all patients who met the criteria for readiness to wean until April 2019. MV was disconnected from the patient, and supplemental oxygen was provided as a blended gas at a flow of 9L/min with less than 40% of inspired oxygen fraction through the T-piece system connected to the endotracheal or tracheostomy tube. In April 2019, our hospital revised the weaning protocol for conducting SBT using inspiratory pressure augmentation rather than T-piece in patients with an endotracheal tube and this novel protocol was implemented in selected patients for a 3-month transition period for feasibility. Then, from July 1, 2019, the revised protocol using inspiratory pressure augmentation for SBT was implemented to all patients who met the criteria for readiness to wean. The patients underwent SBT while attached to the ventilator settings with pressure support of 8 cmH_2_O and PEEP of 0. FiO_2_ maintained the same as before the SBT. The initial attempt was targeted at 30 min for both the T-piece trial and inspiratory pressure augmentation, but the trial was immediately terminated when a sign of SBT failure occurred (Table 2). The patients’ blood pressure, heart rates, respiratory rates, and transcutaneous oxygen saturation were continuously monitored during the trial. When the trial was terminated, arterial blood was obtained for blood gas analysis, and the patient returned to MV with the same ventilator settings as those before the SBT.

SBT failure is defined when the patient had one or more of the following criteria: depressed mental status, signs of increased breathing effort or distress (i.e., agitation and anxiety, diaphoresis, and accessory muscle use), tachypnea (i.e., respiratory rate more than 35/min or increased by ≥ 50%), hemodynamic changes (i.e., cardiac arrhythmias, heart rate of more than 140/min or increased by ≥ 20%, and systolic blood pressure of less than 90 mmHg or more than 180 mmHg or increased by ≥ 20%), hypoxemia (i.e., cyanosis, partial pressure of oxygen in arterial blood of less than 60 mmHg or arterial oxygen saturations of less than 90% on FiO_2_ ≥ 0.4), or hypercapnia (i.e., partial pressure of carbon dioxide in arterial blood of more than 45 mmHg or an increase by ≥ 20% from pre-SBT and pH of less than 7.32 and a decrease in pH ≥ 0.07).

| **Table 2. Criteria for SBT failure** |
| --- |
| Clinical assessment   - Agitation and anxiety - Depressed mental status - Diaphoresis - Cyanosis - Evidence of increasing effort - Increased accessory muscle activity - Facial signs of distress - Dyspnea |
| Objective measurements   - PaO_2_ < 60 mmHg or SaO_2_ < 90% on FiO_2_ ≥ 0.4 - PaCO_2_ > 45 mmHg or an increase in ≥ 20% from pre-SBT and pH < 7.32 and a decrease in pH ≥ 0.07 from pre-SBT - Respiratory rates > 35 breaths/min or increased by ≥ 50% from pre-SBT - Heart rates >140 beats/min or increased by ≥ 20% from pre-SBT - Systolic blood pressure > 180 mmHg or increased by ≥ 20% from pre-SBT, or systolic blood pressure < 90 mmHg - Cardiac arrhythmias |

If the patient successfully passed the SBT, extubation was performed immediately. Followed extubation, oxygen supplement was provided by a nonrebreathing facemask at 9–10 L/min, with an FiO_2_ of 40%. Since July 2017, we have modified the protocol of oxygen therapy following planned extubation using high-flow nasal cannula (HFNC, Optiflow, Fisher and Paykel Healthcare) routinely based on the results of recent trials. The flow was initially set at 30 L/min with an FiO_2_ of 40% and was adjusted according to patient oxygenation requirements. After 24 h, the flow was titrated downward to 25 L/min and changed to a conventional nasal cannula, if tolerable to the patient. Re-intubation was considered when patient had one or more of the extubation failure criteria during 48 hours after extubation (Table 3).

| **Table 3. Criteria for extubation failure** |
| --- |
| - Respiratory rates > 35 breaths/min for 2hrs - Heart rates > 140/min or sustained increase or decrease of ≥ 20% pre-extubation - Clinical signs of respiratory muscle fatigue or increased work of breathing - Inadequate oxygenation: PaO_2_ < 60 mmHg or SaO_2_ < 90% on FiO_2_ ≥ 0.4 - Inadequate ventilation: PaCO_2_ > 45 mmHg or an increase in ≥20% from pre-extubation |

If the patient failed the SBT, MV was resumed, and the multidisciplinary team evaluated the cause of failure, corrected the reversible factors, and repeated screening and SBT the following day. The process of the initial and subsequent SBTs was the same, except for targeting a 120-min period.
