# Supplementary material for: Comparison between pressure support ventilation and T-piece in spontaneous breathing trials
Source: Respir Res. 2022 Feb 7;23:22. doi: 10.1186/s12931-022-01942-w (PMC8822807; doi:10.1186/s12931-022-01942-w)

Additional file 2

**Comparison between pressure support ventilation and T-piece in spontaneous breathing trials**

Soo Jin Na, Ryoung-Eun Ko, Jimyoung Nam, Myeong Gyun Ko, Kyeongman Jeon^,^

**Figure S1. Proportion of prolonged weaning according to comorbidities**


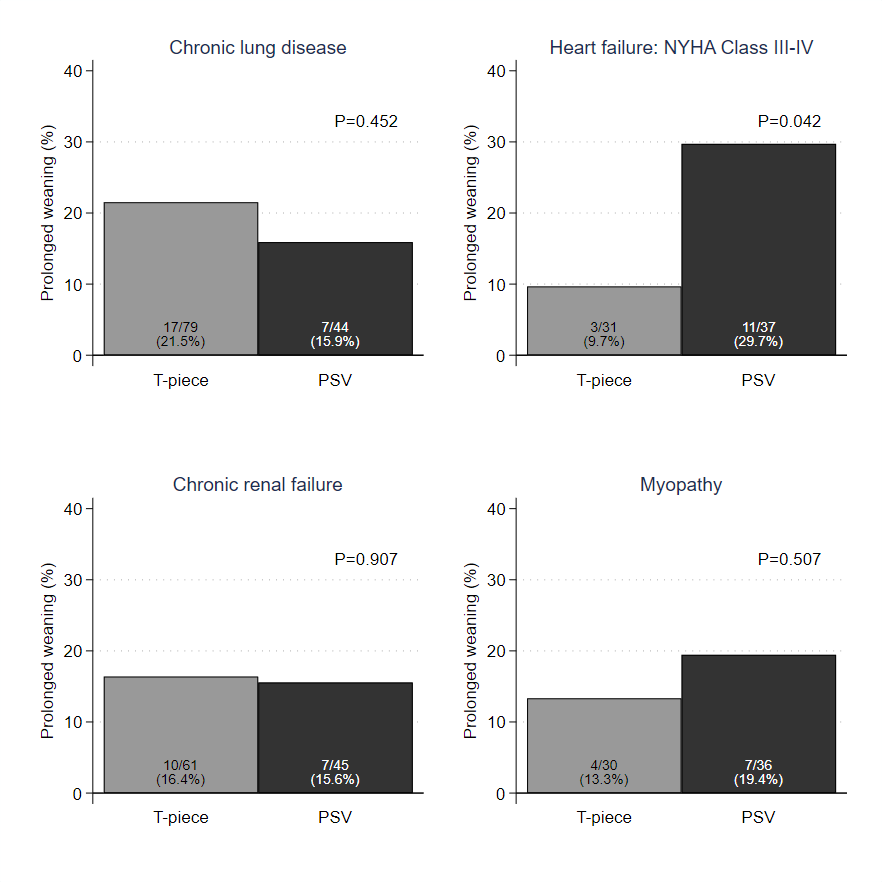

Supplement: Supplementary file 2 — Additional file 2: Figure S1. Proportion of prolonged weaning according to comorbidities. [file 12931_2022_1942_MOESM2_ESM.docx]
