# Supplementary material for: Comparison between pressure support ventilation and T-piece in spontaneous breathing trials
Source: Respir Res. 2022 Feb 7;23:22. doi: 10.1186/s12931-022-01942-w (PMC8822807; doi:10.1186/s12931-022-01942-w)
Supplement: Supplementary file 3 — Additional file 3: Figure S2. Interactions and odds ratios for successful weaning by subgroups. [file 12931_2022_1942_MOESM3_ESM.docx]

Additional file 3

**Comparison between pressure support ventilation and T-piece in spontaneous breathing trials**

Soo Jin Na, Ryoung-Eun Ko, Jimyoung Nam, Myeong Gyun Ko, Kyeongman Jeon^,^

**Figure S2. Interactions and odds ratios for successful weaning by subgroups.**


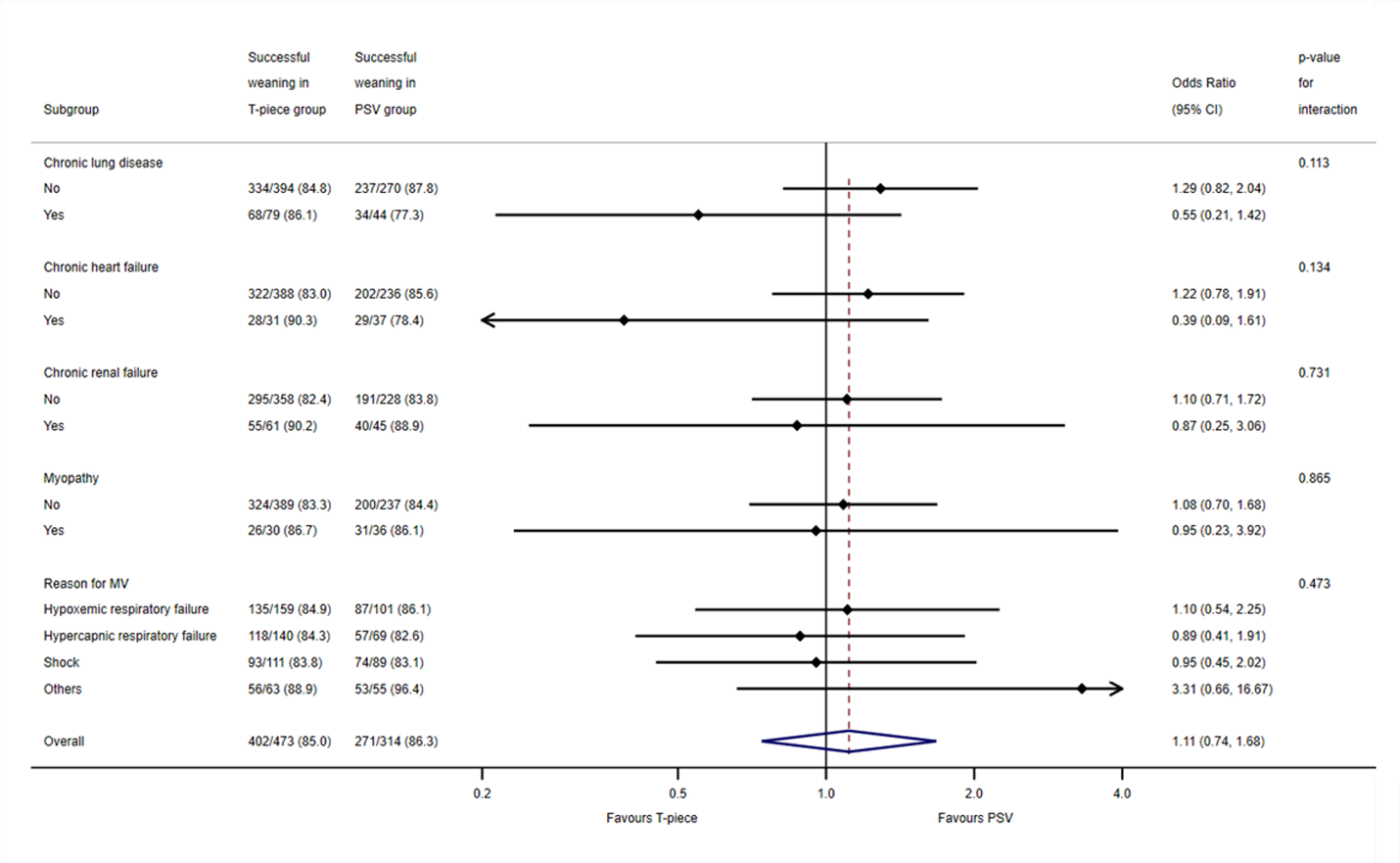


MV = mechanical ventilation
