# Supplementary material for: Comparison between pressure support ventilation and T-piece in spontaneous breathing trials
Source: Respir Res. 2022 Feb 7;23:22. doi: 10.1186/s12931-022-01942-w (PMC8822807; doi:10.1186/s12931-022-01942-w)
Supplement: Supplementary file 4 — Additional file 4: Table S1. Baseline demographic and clinical characteristics of the patient who underwent the first SBT using endotracheal tube (n = 635). Table S2. Characteristics at the day of first spontaneous breathing trial of patient who performed first SBT using endotracheal tube (n = 635). [file 12931_2022_1942_MOESM4_ESM.docx]

Additional file 4

**Comparison between pressure support ventilation and T-piece in spontaneous breathing trials**

Soo Jin Na, Ryoung-Eun Ko, Jimyoung Nam, Myeong Gyun Ko, Kyeongman Jeon^,^

**Table S1. Baseline demographic and clinical characteristics of the patient who underwent the first SBT using endotracheal tube (n = 635).**

|  | T-piece group  (n = 375) | PSV group  (n = 260) | *P*-value |
| --- | --- | --- | --- |
| Age, years | 67 (57–75) | 67 (56–76) | 0.975 |
| Male | 239 (63.7) | 167 (64.2) | 0.898 |
| Body mass index, kg/m^2^ | 22.9 (20.2–25.6) | 22.8 (19.8–25.9) | 0.919 |
| Comorbidities |  |  |  |
| Chronic obstructive pulmonary disease | 27 (8.2) | 20 (8.9) | 0.765 |
| Asthma | 6 (1.8) | 3 (1.3) | 0.745 |
| Interstitial lung disease | 7 (2.1) | 10 (4.5) | 0.118 |
| Heart failure: NYHA classes III–IV | 26 (7.9) | 30 (13.4) | 0.036 |
| Chronic renal failure | 55 (16.7) | 33 (14.7) | 0.531 |
| Liver cirrhosis: Child-Pugh Class C | 9 (2.7) | 9 (4.0) | 0.404 |
| Solid/hematologic malignancy | 205 (62.3) | 147 (65.6) | 0.426 |
| Myopathies/Neuropathies | 20 (6.1) | 20 (8.9) | 0.204 |
| Dementia | 106 (28.3) | 69 (26.5) | 0.632 |
| Major reason for MV |  |  | 0.032 |
| Hypoxemic respiratory failure | 121 (32.3) | 76 (29.2) |  |
| Hypercapnic respiratory failure | 108 (28.8) | 54 (20.8) |  |
| Shock | 93 (24.8) | 82 (31.5) |  |
| Surgery | 2 (0.5) | 5 (1.9) |  |
| Others ^a^ | 51 (13.6) | 43 (16.5) |  |

Values are interquartile ranges or n (%).

^a^ Others include airway protection, neurological impairment, and metabolic causes.

MV, mechanical ventilation; NYHA, New York Heart Association; PSV, pressure support ventilation.

**Table S2. Characteristics at the day of first spontaneous breathing trial of patient who performed first SBT using endotracheal tube (n = 635).**

|  | T-piece group  (n = 375) | PSV group  (n = 260) | *P*-value |
| --- | --- | --- | --- |
| Duration of MV before first spontaneous breathing trial, days | 4 (3–6) | 5 (3–7) | 0.026 |
| Medical management |  |  |  |
| Vasoactive drug | 70 (19.6) | 25 (9.8) | 0.001 |
| Sedatives | 135 (37.8) | 106 (41.7) | 0.329 |
| Opioid | 281 (78.7) | 179 (70.5) | 0.020 |
| Steroid | 163 (45.7) | 112 (44.1) | 0.702 |
| Diuretics | 140 (39.2) | 99 (39.0) | 0.952 |
| Renal replacement therapy | 50 (14.0) | 49 (19.3) | 0.081 |
| RASS score |  |  | 0.581 |
| RASS −1–+1 | 271 (76.1) | 202 (79.5) |  |
| RASS < −1 | 73 (20.5) | 46 (18.1) |  |
| RASS > +1 | 12 (3.3) | 6 (2.4) |  |
| SOFA scores | 7 (5–10) | 7 (5–10) | 0.367 |
| Setting of MV |  |  |  |
| Mode |  |  | 0.251 |
| Volume controlled ventilation | 0 (0.0) | 1 (0.3) |  |
| Pressure controlled ventilation | 48 (10.6) | 27 (8.8) |  |
| Synchronized intermittent mandatory Ventilation | 0 (0.0) | 1 (0.3) |  |
| Pressure support ventilation | 406 (89.4) | 278 (90.6) |  |
| Peak inspiratory pressure, cmH_2_O | 16 (14–18) | 16 (14–18) | 0.122 |
| Respiratory rate, breath/min | 17 (14–21) | 18 (14–22) | 0.533 |
| PEEP, cmH_2_O | 5 (5–5) | 5 (5–5) | 0.832 |
| Monitored Vt/PBW, mL/kg | 8.2 (6.6–10.3) | 7.4 (6.0–9.4) | 0.004 |
| FiO_2_, % | 30 (30–40) | 30 (25–40) | 0.007 |
| PaO_2_/FiO_2_ ratio | 291 (232–378) | 303 (235–394) | 0.241 |
| Arterial blood gas |  |  |  |
| pH | 7.454 (7.418–7.488) | 7.471 (7.436–7.502) | 0.002 |
| PaCO_2_, mmHg | 35.4 (31.0–40.7) | 33.8 (28.6–39.3) | 0.065 |
| PaO_2_, mmHg | 92.2 (79.9–106.8) | 90.0 (79.8–103.3) | 0.558 |
| SaO_2_, % | 97.0 (95.9–98.4) | 97.0 (95.8–98.0) | 0.168 |
| Lactate, mmol/L | 1.69 (1.14–2.33) | 2.01 (1.25–2.49) | 0.436 |

Values are interquartile range or n (%).

FiO_2_ indicates fraction of inspired oxygen; MV, mechanical ventilation; PaCO_2_, partial pressure of carbon dioxide in arterial blood; PaO_2_, partial pressure of oxygen in arterial blood; PBW, predicted body weight; PEEP, positive end-expiratory pressure; PSV, pressure support ventilation; SaO_2_, arterial oxygen saturations; SOFA, sequential organ failure assessment; RASS, Richmond Agitation–Sedation Scale; VT, tidal volume.
